# Current comparator for both AC and DC ratio measurements with 10^− 8^-level type-a uncertainty

**DOI:** 10.1038/s41598-026-58868-2

**Published:** 2026-08-11

**Authors:** Hidekazu Muramatsu, Yuta Kainuma, Hiromitsu Kato, Norihiko Sakamoto, Tatsuji Yamada, Chiharu Urano, Hiroshi Abe, Shinobu Onoda, Takeshi Ohshima, Yuji Hatano, Mutsuko Hatano, Nobu-Hisa Kaneko, Yasutaka Amagai, Takayuki Iwasaki

**Affiliations:** 1 National Metrology Institute of Japan (NMIJ), National Institute of Advanced Industrial Science and Technology (AIST), Ibaraki, Japan; 2 Global Research and Development Center for Business by Quantum-AI Technology (G-QuAT), National Institute of Advanced Industrial Science and Technology (AIST), Ibaraki, Japan; 3 Advance Power Electronics Research Center, National Institute of Advanced Industrial Science and Technology (AIST), Ibaraki, Japan; 4 National Institutes for Quantum Science and Technology (QST), Gunma, Japan; 5 Department of Material Science, Tohoku University, Miyagi, Japan; 6 Department of Electrical and Electronic Engineering, School of Engineering, Institute of Science Tokyo, Tokyo, Japan

**Keywords:** Engineering, Materials science, Physics

## Abstract

Accurate measurements of alternating current (AC) and direct current (DC) ratios are fundamental to electric power metrology. However, conventional current comparators for AC and DC typically rely on distinct technologies—electromagnetic induction for AC and superconducting quantum interference devices for DC. This technological divide leads to a fragmented and complex traceability system. Bridging this gap is critical for developing unified current standards that meet the demands of temperature power technologies.　In this work, we present a compact, room-temperature AC/DC current comparator that integrates a diamond-based magnetometer using nitrogen-vacancy centers. The device achieves a type-A uncertainty at $\:{10}^{-8}$ level, while the expanded uncertainty is at $\:{10}^{-6}$to $\:{10}^{-7}\:$level, for both AC and DC signals and supports a system bandwidth up to $\:300\:\text{ }\mathrm{H}\mathrm{z}$, without the need for cryogenics. It is comparable to the performance of typical AC comparators, and provides a compact and room-temperature operating platform for further performance improvement. This unified, cryogenics-free solution not only enhances precision and versatility but also expands the applicability of the system to DC resistance bridges in quantum electrical standards.

**Supplementary Information:**

The online version contains supplementary material available at 10.1038/s41598-026-58868-2.

## Introduction

Distributed energy sources such as solar and wind, along with power electronic devices, are increasingly being integrated into modern power grids. These advancements introduce not only conventional power-line frequency currents $\:(50\:\text{ }\mathrm{H}\mathrm{z}/60\:\text{ }\mathrm{H}\mathrm{z})$ but also harmonic and direct currents (DC). Given that current transformers are widely used to scale high currents to levels suitable for measurement and protection in industrial settings, accurate current ratio measurements across a broad frequency range are essential for reliable grid monitoring.

Traditionally, current ratio measurements rely on current comparators^1,2^, which determine the ratio between two currents by detecting the null magnetic flux in a high-permeability core. Conventional alternating current comparators^3,4^ (ACCs), which use detection windings based on electromagnetic induction, serve as primary standards in several national metrology institutes (NMIs), achieving accuracies on the order of $\:{10}^{-6}$. While this level of accuracy suffices for many applications, further improvements are limited by the core’s physical properties and the inherent constraints of electromagnetic detection. For DC applications, cryogenic current comparators^2,5,6^ (CCCs) or DC comparators^7–11^ (DCCs), which use superconducting quantum interference devices^12–14^ (SQUIDs) or fluxgate sensors, can reach accuracies of approximately $\:{10}^{-9}$. CCCs for AC applications, such as AC-bridges, have also been reported, demonstrating accuracies on the order of $\:{10}^{-8}$[15].

However, a significant limitation of existing metrology systems lies in their fragmentation: AC and DC measurements require distinct comparator technologies. This separation complicates the calibration infrastructure and reduces measurement flexibility. Furthermore, conventional current comparators often involve bulky windings—up to * 500* turns—and, in some cases, the need for cryogenic cooling. A single, compact device capable of performing accurate current ratio measurements across both AC and DC regimes without cryogenics would streamline traceability and broaden the applicability of high-precision current metrology in industrial environments.

To overcome these challenges, we leverage nitrogen-vacancy (NV) centers in diamond^16–19^, which have emerged as powerful candidates for quantum magnetic-field sensing. NV centers allow magnetic field detection across a wide frequency range—from DC up to several $\:\mathrm{M}\mathrm{H}\mathrm{z}$^20–22^ through optical interrogation of their electron spin states. They offer a broad dynamic range near zero magnetic field²³, high sensitivity without the need for cryogenic cooling, and resilience to temperature fluctuations, making them ideally suited as null detectors for both AC and DC current ratio measurements. While NV-based magnetometers have previously been used for magnetic field sensing, their use in precision current ratio metrology—particularly as unified AC/DC current comparators—has not yet been demonstrated.

In this study, we developed a compact current comparator integrating a diamond quantum sensor and successfully demonstrated accurate current ratio measurements with a type-A uncertainty at the * 10⁻⁸* level across both AC and DC regimes.　To achieve this goal, a low-noise and highly sensitive NV-diamond magnetometer was developed and embedded in the air gap of a magnetic core, eliminating the need for large detection windings and massive magnetic cores while improving the sensitivity. This work represents a significant step toward unifying AC and DC　current ratio measurements in a room-temperature platform, thereby simplifying electrical metrology traceability and expanding the applicability of quantum sensors in precision instrumentation.

**Fig. 1:**
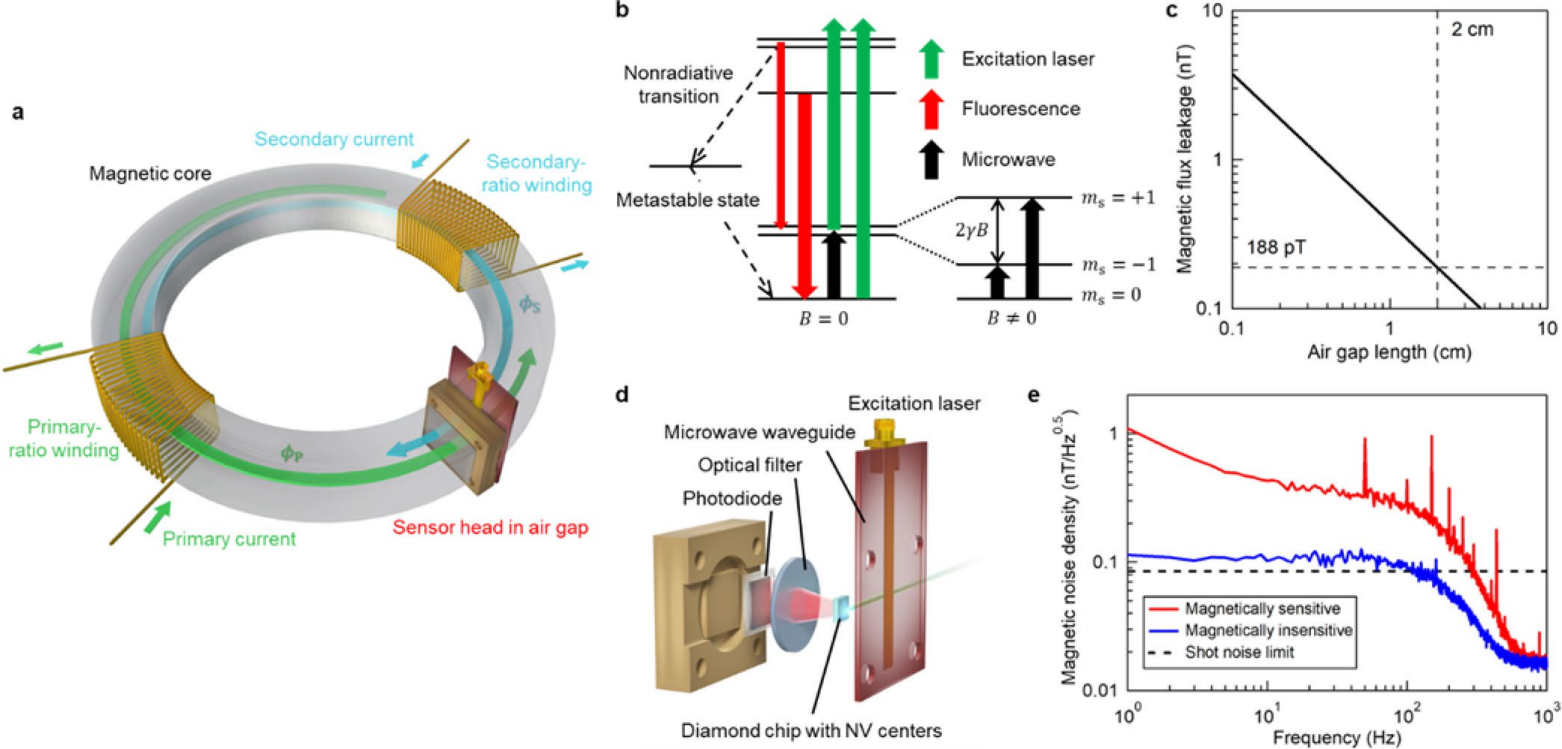
Precision current ratio measurement using a solid-state quantum sensor. **a**, Schematic of an NV-diamond-based current comparator. The primary- and secondary-ratio windings are wound around the air-gapped magnetic core. Although the figure depicts the two windings as being concentrated on diametrically opposite localized regions of the core, they are in fact uniformly distributed over the entire magnetic core. The diamond quantum sensor placed in the air gap of the core measures the residual magnetic flux produced by the currents in the ratio windings. $\:{\phi\:}_{\mathrm{P}}$ and $\:{\phi\:}_{\mathrm{S}}$ are magnetic fluxes generated by the currents. **b**, Energy level diagram of the NV center. The $\:{m}_{\mathrm{s}}=\pm\:1$ ground states are split by an external magnetic field with the width proportional to the magnitude of the magnetic field * B* and gyromagnetic ratio $\:\gamma\:$. **c**, Calculated magnetic flux in the air gap as a function of the air-gap length. **d**, Schematic of the sensor head of the diamond magnetometer. The sensor head includes a (111)-oriented diamond chip placed on a microwave waveguide, an optical filter, and a photodiode within a width of approximately 1 cm. **e**, Magnetic noise spectrum of the present system. The red solid line shows the magnetic noise spectral density under magnetically sensitive conditions. The blue solid line shows the magnetic noise spectrum excited by microwaves at non-resonant frequencies. The dotted line represents the calculated photon shot-noise limit.

## Results and discussions

Design of a current comparator measuring both AC and DC current ratios.

As part of our effort to develop a current comparator capable of accurately measuring both AC and DC current ratios at a level suitable for primary standards, we began by analyzing the structure and operating principle of conventional devices. A traditional alternating current comparator (ACC) determines the ratio between two currents—applied to the primary and secondary windings—wound around a high-permeability toroidal magnetic core (without an air gap). These currents generate opposing magnetic fluxes within the core, which ideally cancel each other out. The balance point, or null condition, is detected by a detection winding that measures the residual magnetic flux in the core. Typically, such magnetic cores have a diameter of around 30 cm, and the detection winding may consist of up to 500 turns, resulting in a total weight of approximately 20 kg.

A nickel-iron-based alloy (permalloy) is commonly used as the magnetic core material due to its high magnetic permeability and low coercivity, making it ideal for precision magnetic applications. Following the configuration of a conventional ACC, we designed our current comparator to operate at the most fundamental current ratio—namely, a one-to-one ratio. The developed one-to-one current comparator consists of an air-gapped magnetic core integrated with a low-noise diamond magnetometer, along with primary and secondary windings (Fig. 1a). The residual magnetic flux in the air gap is measured by the diamond magnetometer, which incorporates an ensemble of nitrogen-vacancy (NV) centers in diamond.

We employed continuous-wave optically detected magnetic resonance (cw-ODMR) for magnetic field detection. In cw-ODMR, the energy splitting between the $\:{m}_{\mathrm{s}}=\pm\:1\:$spin states (Fig. 1b), where $\:{m}_{\mathrm{s}}$ is the magnetic quantum number, is inferred from the fluorescence intensity. The NV spin states are optically initialized using a continuous-wave green laser and manipulated by applying microwaves at their resonant frequencies. Although the resonance frequencies are influenced by both magnetic field and temperature, temperature-induced shifts appear as a common-mode contribution, whereas the magnetic field manifests as a differential splitting. Therefore, the residual magnetic flux can be precisely determined by measuring the frequency difference between the two resonances corresponding to the Zeeman-induced splitting.

**Fig. 2:**
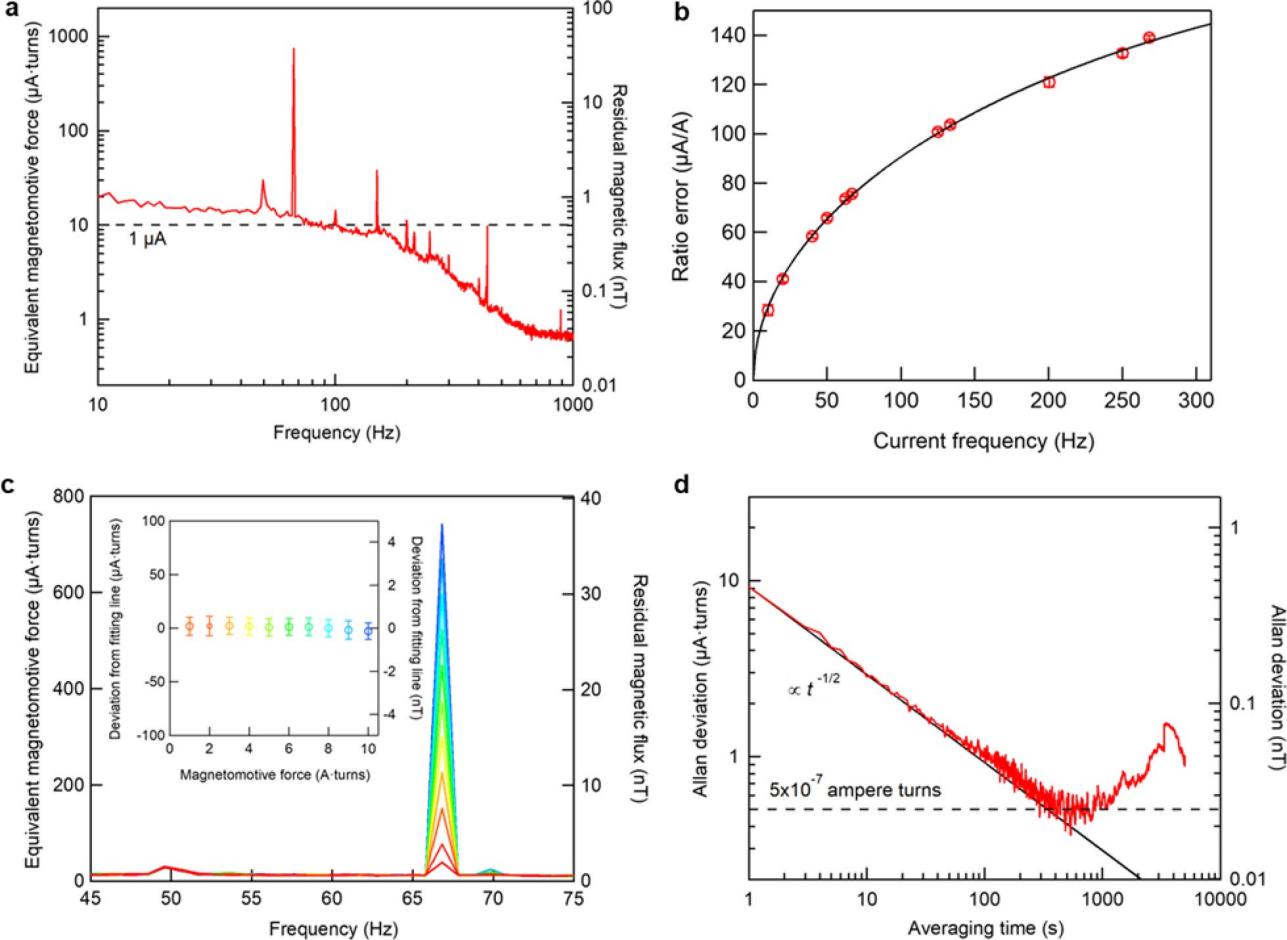
The performance of the developed current comparator for AC ratio measurements. **a**, Amplitude spectra of the developed one-to-one current comparator obtained by a one-second measurement at a current frequency of $\:67\:\text{ }\mathrm{H}\mathrm{z}$. The left and right axes represent the equivalent current difference and residual magnetic flux, respectively. A clear peak appears at the expected frequency of $\:67\:\text{ }\mathrm{H}\mathrm{z}$. The noise floor of the present system is approximately $\:1\:\text{ }{\upmu\:}\mathrm{A}$ (denoted by the dotted line in **a**). **b**, Equivalent current difference as a function of frequency. The solid line represents the fitting curve based on the model of the hysteresis loss and the eddy current loss. **c**, The equivalent current difference measured by the diamond quantum sensor is proportional to the amplitude of the input current, as shown in the inset of **c**. Each dot in the inset represents the deviation between the fitted line and the peak value of the corresponding color spectrum. Each residual flux was averaged 100 times, and the error bars, which represent the standard deviation of the mean. The slope of the solid line represents the ratio error of the current comparator at $\:67\:\text{ }\mathrm{H}\mathrm{z}$. **d**, Allan deviation at an amplitude of $\:1\:\text{ }\mathrm{A}$ and a frequency of $\:67\:\text{ }\mathrm{H}\mathrm{z}$. The red solid line represents the theoretical curve assuming white noise. The horizontal dotted line denotes the detectable current limit, corresponding to $\:50\:\text{ }\mathrm{n}\mathrm{A}$[24].

To assess the sensitivity requirements for the diamond magnetometer, we calculated the expected magnetic flux amplitude within the core using a magnetic equivalent circuit model for a core with a 2 cm air gap.

The analysis revealed that the magnetic flux, which depends on factors such as relative permeability, cross-section area of the magnetic core, and the length of the magnetic path of the core, varies with the air-gap length (Fig. 1c). In the low frequency range, uncertainties of ACCs are dominated by the resolution of the ampere-turn balance, defined as the ability to resolve the residual magnetic flux, which is ultimately limited by the sensitivity of the null detector. To achieve a resolution comparable to that of the primary standard at NMIJ—$\:3\times\:{10}^{-6}\:\text{ }\mathrm{a}\mathrm{m}\mathrm{p}\mathrm{e}\mathrm{r}\mathrm{e}\:\mathrm{t}\mathrm{u}\mathrm{r}\mathrm{n}\mathrm{s}$ corresponding to a relative uncertainty of $\:1.7\times\:{10}^{-6}$—for our current comparator, our calculations indicate that the diamond magnetometer must detect a residual magnetic flux of approximately $\:188\:\text{ }\mathrm{p}\mathrm{T}$ in the $\:2\:\text{ }\mathrm{c}\mathrm{m}$ air gap at a current amplitude of $\:1\:\text{ }\mathrm{A}$ (see Methods).

A schematic of the sensor head of the NV-based magnetometer is shown in Fig. 1d. The design integrates a (111)-oriented diamond directly onto a microwave waveguide, allowing compact placement within the air gap of the magnetic core without requiring external optics or bulky components. The sensor head consists of a (111)-oriented diamond chip containing an ensemble of NV centers, positioned on a microwave waveguide for spin manipulation. An optical filter is used to block background light, including the excitation laser, and a photodiode detects the NV red fluorescence. The diamond’s (111) surface is oriented perpendicular to the direction of the residual magnetic flux in the air gap. The excitation laser is introduced from one of the side faces of the diamond chip. All components are integrated within a width of approximately $\:1\:\text{ }\mathrm{c}\mathrm{m}$, enabling the sensor to fit inside the $\:2\:\text{ }\mathrm{c}\mathrm{m}$ air gap^25^. The magnetic core has an outer diameter of $\:10\:\text{ }\mathrm{c}\mathrm{m}$, an inner diameter of $\:6\:\text{ }\mathrm{c}\mathrm{m}$, and a thickness of $\:2\:\text{ }\mathrm{c}\mathrm{m}$—roughly one-third the size of typical magnetic cores used in conventional ACCs. Its weight is approximately 1 kg, which is one-twentieth that of magnetic cores typically used in conventional ACCs (see Methods).

To evaluate whether the diamond magnetometer can satisfy the requirement of $\:188\:\text{ }\mathrm{p}\mathrm{T}$, we measured the noise spectra under both magnetically sensitive and magnetically insensitive conditions (Fig. 1e). While no current was applied to the primary and secondary ratio windings, a DC current was supplied to an auxiliary winding to generate an offset DC magnetic field. This offset was necessary to avoid the nonlinear response region near zero magnetic field in the ODMR spectrum. To measure the magnetic field magnitude, we employed a time-domain multiplexed frequency modulation technique^24^, which enables simultaneous tracking of the two Zeeman-split resonance frequencies.

As shown by the blue solid line in Fig. 1e, setting the system to a non-resonant microwave frequency of $\:2\:\text{ }\mathrm{G}\mathrm{H}\mathrm{z}$ resulted in a magnetically insensitive noise floor of approximately $\:100\:\text{ }\mathrm{p}\mathrm{T}/{\mathrm{H}\mathrm{z}}^{0.5}$ in the $\:10\:\text{ }\mathrm{H}\mathrm{z}$ to $\:100\:\text{ }\mathrm{H}\mathrm{z}$ frequency range. Under these magnetically insensitive conditions, the noise floor was primarily limited by photon shot noise, estimated to be $\:85\:\text{ }\mathrm{p}\mathrm{T}/{\mathrm{H}\mathrm{z}}^{0.5}$. In contrast, the red solid line shows the magnetic noise spectrum under magnetically sensitive conditions, where the noise floor increased to approximately $\:300\:\text{ }\mathrm{p}\mathrm{T}/{\mathrm{H}\mathrm{z}}^{0.5}$ at the fundamental power-line frequencies.

Since this $\:300\:\text{ }\mathrm{p}\mathrm{T}/{\mathrm{H}\mathrm{z}}^{0.5}$ noise floor is dominated by random noise sources, the magnetic sensitivity of the system improves with longer integration times, scaling inversely with the square root of the integration time.

Under this assumption, the target detection limit of $\:188\:\text{ }\mathrm{p}\mathrm{T}$— corresponding to a relative uncertainty of $\:1.7\times\:{10}^{-6}$ in a $\:2\:\text{ }\mathrm{c}\mathrm{m}$ air gap—can be achieved by integrating for approximately $\:2.5\:\text{ }\mathrm{s}$, being comparable with a standard measurement time in electrical standards. This suggests that the developed diamond magnetometer is capable of supporting AC current ratio measurements with a resolution comparable to the $\:{10}^{-6}$ level of accuracy typically attained by conventional current comparators used as national standard at NMIs.

### Evaluation of ratio error and stability

We performed AC current ratio measurements to evaluate the performance of the developed current comparator. For this purpose, the primary and secondary ratio windings, each with * 10* turns, were connected in series. In an ideal current comparator, the current ratio is solely determined by the turn ratio of the windings. However, practical deviations—due to leakage flux, stray capacitance, and other parasitic effects—introduce what is known as ratio error¹.

To quantify this ratio error, the residual magnetic flux in the air gap was measured using the NV-diamond magnetometer. A constant DC magnetic field offset was applied to position the NV resonance within its linear response regime. An AC current was then supplied to the series-connected ratio windings, generating a magnetic flux detectable by the sensor. The resulting time-domain magnetic field signal—comprising the AC component, noise, and DC offset—was analyzed using a discrete Fourier transform (DFT) to extract the flux component at the excitation frequency.

To assess performance at a representative power-line frequency, we measured the amplitude spectrum at a fixed input current of $\:1\:\text{ }\mathrm{A}$ corresponding to $\:10\:\text{ }\mathrm{a}\mathrm{m}\mathrm{p}\mathrm{e}\mathrm{r}\mathrm{e}\:\mathrm{t}\mathrm{u}\mathrm{r}\mathrm{n}\mathrm{s}$ (Fig. 2a). The excitation frequency was set to $\:67\:\text{ }\mathrm{H}\mathrm{z}$ to avoid interference from common $\:50\:\text{ }\mathrm{H}\mathrm{z}/60\:\text{ }\mathrm{H}\mathrm{z}$ power-line noise and its harmonics, which can degrade low-frequency magnetic measurements. The equivalent difference between magnetomotive forces was determined using a conversion coefficient relating the residual magnetic flux to magnetomotive force deviation at $\:67\:\text{ }\mathrm{H}\mathrm{z}$ (see Methods). As shown in the amplitude spectrum, a clear peak was observed at the expected frequency of $\:67\:\text{ }\mathrm{H}\mathrm{z}$. The measured noise floor of the current comparator system was approximately $\:1.0\times\:{10}^{-5}\:\text{ }\mathrm{a}\mathrm{m}\mathrm{p}\mathrm{e}\mathrm{r}\mathrm{e}\:\mathrm{t}\mathrm{u}\mathrm{r}\mathrm{n}\mathrm{s}$, denoted by the dotted line, as shown in Fig. 2a. To investigate how the measured current ratio varies with the frequency of the input current, we conducted residual magnetic flux measurements across several frequencies. The measured ratio error as a function of input frequency at a fixed current amplitude of $\:1\:\text{ }\mathrm{A}$ was evaluated over a range of frequencies (Fig. 2b). The system exhibited a bandwidth of $\:300\:\text{ }\mathrm{H}\mathrm{z}$, which was limited by the low-pass filter setting of the lock-in amplifier. The observed frequency response may result from eddy current losses and hysteresis effects in the high-permeability magnetic core material^26^.

Next, we examined whether the residual magnetic flux—and thus the difference between equivalent magnetomotive forces—is linearly proportional to the input current amplitude. To do so, we acquired magnetic field spectra while systematically varying the current amplitude. Both the measured difference between equivalent magnetomotive forces and corresponding residual flux scaled linearly with input current within the error bars (Fig. 2c), confirming the system’s stable and linear performance over a range of current amplitudes. The error bars represent the standard deviation of the mean obtained from 100 times measurements.

Under typical operating conditions, we quantified the ratio error in the current comparator. At an input amplitude of $\:1\:\text{ }\mathrm{A}$ and $\:67\:\text{ }\mathrm{H}\mathrm{z}$, the measured difference between equivalent magnetomotive forces was $\:7.6\times\:{10}^{-4}\:\text{ }\mathrm{a}\mathrm{m}\mathrm{p}\mathrm{e}\mathrm{r}\mathrm{e}\:\mathrm{t}\mathrm{u}\mathrm{r}\mathrm{n}\mathrm{s}$, yielding a ratio error of $\:76\:\text{ }{\upmu\:}\mathrm{A}/\mathrm{A}$. Although this does not meet the stringent $\:1\:\text{ }{\upmu\:}\mathrm{A}/\mathrm{A}$ requirement typically expected in metrological applications, the observed error reflects performance under the present experimental conditions and does not represent the fundamental limit of the developed system. Given the stability of the error over time and varying conditions, it is possible to correct for it through calibration, enabling the comparator to function as a traceable measurement instrument in practical applications.

To evaluate the stability associated with the ratio error, we analyzed the time dependence of the signal-to-noise ratio (SNR) using Allan deviation. The Allan deviation of the mean was calculated for various averaging times * t* (Fig. 2d) to assess the statistical convergence behavior and the attainable precision limits of the current measurements. A clear trend of decreasing Allan deviation with increasing averaging time was observed, reaching a minimum value of $\:25\:\text{ }\mathrm{p}\mathrm{T}$ before increasing again. The observed $\:{t}^{-1/2}$ time dependence confirms that the noise in the system is dominated by uncorrelated random noise.

At $\:67\:\text{ }\mathrm{H}\mathrm{z}$, the Allan deviation reached a minimum of $\:5\times\:{10}^{-7}$ ampere turns at an averaging time of $\:500\:\text{ }\mathrm{s}$. Therefore, the type-A uncertainty (* k=1*) ²⁷ at an amplitude of $\:1\:\text{ }\mathrm{A}$ and a frequency of $\:67\:\text{ }\mathrm{H}\mathrm{z}$ could be derived as $\:50\:\text{ }\mathrm{n}\mathrm{A}/\mathrm{A}$, corresponding to a relative uncertainty of $\:5\times\:{10}^{-8}$. Precision current comparators used in NMIs typically require a relative uncertainty of about $\:1\:\text{ }{\upmu\:}\mathrm{A}/\mathrm{A}$. The type-A uncertainty achieved with the present device is already below this threshold, indicating that the present device has the potential to satisfy the primary-standard requirement.

To understand how precisely our diamond magnetometer can measure tiny electrical currents, we first examined what sources of error can influence the measurement. For this reason, the uncertainty evaluation must consider not only the statistical variation captured by the Allan-deviation analysis but also other contributions originating from the measurement setup itself.

In particular, in our present experiment setup, we purposely did not use a compensation current^1^, meaning that the sensor directly observed the small magnetic flux that remained inside the magnetic core. This approach makes the measurement principle intuitive, but it also means that several different factors can affect the final accuracy.

To clarify how accurately the diamond magnetometer can determine electrical current, we organized the possible sources of uncertainty into three categories based on their physical origin: (1) uncertainties in the magnetic-flux measurement, (2) uncertainties introduced by the current source, and (3) uncertainties in the conversion coefficients that relate magnetic flux to current.

First, the magnetic-flux uncertainty arises from the noise floor of the voltage-noise spectrum, which sets the smallest detectable magnetic signal. In addition, the conversion from magnetic flux to field requires the gyromagnetic ratio of the NV center, whose known accuracy introduces a small additional contribution^28^.

Second, the current source contributes to uncertainty through the output stability and its specified absolute accuracy.

Third, the conversion coefficient between magnetic flux and current is a dominant contribution to overall uncertainties. This contribution becomes larger when the residual magnetic flux is large, but it can be greatly reduced by introducing a compensation current that drives the flux closer to zero.

By combining these contributions with a square root of sum, we obtain an expanded uncertainty of approximately $\:1.2\times\:{10}^{-6}\:\text{ }\mathrm{A}/\mathrm{A}\:(k=2)$, where *k* is a coverage factor corresponding to 95% level of confidence (Table 1). This value reaches the level required for a primary standard.

**Table 1:** Uncertainty budget for the AC ratio measurement.

Source of uncertainty	Relative standard uncertainty
Type-A uncertainty (Allan deviation)	$\:5.0\times\:{10}^{-8}$
Magnetic flux measurement	$\:4.1\times\:{10}^{-8}$
Current source	$\:6.3\times\:{10}^{-8}$
Flux-current conversion coefficient	$\:8.7\times\:{10}^{-7}$
Combined standard uncertainty	$\:8.8\times\:{10}^{-7}$
Expanded uncertainty (* k=2*)	$\:1.8\times\:{10}^{-6}$

In addition to high precision, the developed current comparator offers advantages in size and integration. Calculations based on a magnetic equivalent circuit model indicate that sensitivity does not degrade with reduced core size. The model suggests that the core can be miniaturized to at least one-tenth of its current dimensions without compromising performance.

In summary, the developed current comparator achieves an expanded uncertainty of $\:1.8\times\:{10}^{-6}\:\text{ }\mathrm{A}/\mathrm{A}$ for AC, being likely to be satisfying the uncertainty requirements for a primary AC current standard. The reduction of the dominant systematic contributions using a zero-flux compensation scheme is left for future work. With its compact design and strong potential for further miniaturization, the system offers a promising pathway toward next-generation current metrology.

### Statistical analysis in DC ratio measurements

**Fig. 3:**
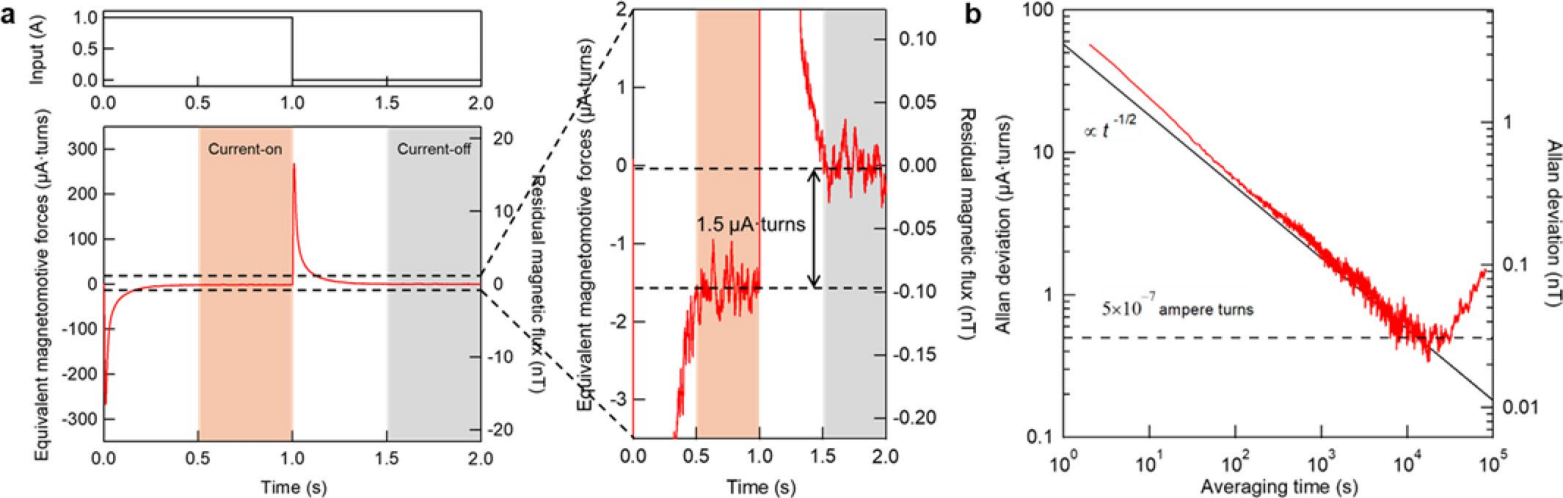
The performance of the developed current comparator for DC ratio measurements. **a**, Time-domain signal of the residual magnetic flux. A square signal with a width of $\:1\:\text{ }\mathrm{s}$ was applied to the ratio windings, and the residual magnetic flux was calculated as the difference between the signals in the current-on and current-off states except for the transient parts. The right figure shows an enlarged view of the near zero nanotesla region of the left figure. The signal of the residual flux was shifted so that the average value in the current-off state was zero. **b**, Allan deviation at a current of $\:1\:\text{ }\mathrm{A}$. The red solid line represents the theoretical curve assuming white noise. The horizontal dotted line denotes the detectable current limit, corresponding to $\:50\:\text{ }\mathrm{n}\mathrm{A}$.

To evaluate the versatility of the developed current comparator, we extended the measurements to DC conditions. Accurate operation under DC excitation is critical for ensuring traceability to DC current ratio standards and for verifying the comparator applicability to both AC and DC metrological scenarios. The measurement configuration of the DC ratio measurements is the same with that of the AC ratio measurements except measurement sequence. Unlike AC measurements—where the residual magnetic flux component at the excitation frequency can be isolated via DFT—DC measurements require a different approach, as the residual flux cannot be separated from constant background fields using frequency-domain techniques. To overcome this, we applied a square-wave current signal with a duration of $\:1\:\text{ }\mathrm{s}$ to the ratio windings, which were connected in series with 10 turns each, as in the AC measurements (top left panel, Fig. 3a). The residual magnetic flux was determined by comparing the NV magnetometer signals during the current-on and current-off states, excluding the transient periods of $\:500\:\text{ }\mathrm{m}\mathrm{s}$, as indicated by the shaded regions in Fig. 3a. The bottom left panel of Fig. 3a shows the detected magnetic flux when a $\:1\:\text{ }\mathrm{A}$ DC current was applied. The DC ratio error of the developed current comparator was measured to be $\:150\:\text{ }\mathrm{n}\mathrm{A}/\mathrm{A}$ $\:(1.5\times\:{10}^{-7})$, based on a residual magnetic flux of approximately $\:95\:\text{ }\mathrm{p}\mathrm{T}$ corresponding to a current difference of $\:150\:\text{ }\mathrm{n}\mathrm{A}$. This performance is comparable to that of conventional.

DCCs, which typically achieve ratio errors at the $\:1\times\:{10}^{-7}$ level, indicating that the developed device meets the requirements for high-accuracy DC current ratio measurements. Although the error does not yet reach the $\:{10}^{-9}$ level attained by CCCs, it is sufficient for several metrological and industrial calibration applications. However, notably, the observed ratio error is condition-dependent and does not represent the ultimate value of the system. Further improvements in shielding and noise suppression may enable even lower ratio errors, approaching those of established DC standards.

The Allan deviation of the DC measurements is shown in Fig. 3b. As in the AC case, the Allan deviation decreased with a $\:{t}^{-1/2}$ time dependence, confirming that uncorrelated random noise was dominant. The minimum Allan deviation reached approximately $\:30\:\text{ }\mathrm{p}\mathrm{T}$, corresponding to a detectable current difference of $\:5\times\:{10}^{-7}$ ampere turns. This yields a type-A uncertainty of $\:50\:\text{ }\mathrm{n}\mathrm{A}/\mathrm{A}$ at $\:1\:\text{ }\mathrm{A}$ input current, corresponding to a relative uncertainty of $\:5\times\:{10}^{-8}$. This value is significantly lower than those reported for other NV-diamond-based sensors operating under ambient conditions, which typically exceed $\:200\:\text{ }{\upmu\:}\mathrm{A}$^29^–^31^.

In the DC measurements, the uncertainty budget was constructed from the magnetic-flux measurement, the current source, and the conversion coefficient. The uncertainty structure in DC measurements mirrors that of the AC case, because the measurement principle—detecting magnetic flux induced by the current—remains unchanged, as summarized in Table 2. By combining these contributions, the resulting expanded uncertainty was $\:2.7\times\:{10}^{-7}\:\text{ }\mathrm{A}/\mathrm{A}$. While CCCs offer sub-nanoampere resolution, they require complex, cryogenic infrastructure. In contrast, the present system achieves nanoampere-level resolution in a compact, room-temperature setup. However, due to low-frequency noise under static conditions (Fig. 1e), achieving this resolution required an averaging time approximately 40 times longer than that needed for AC measurements.

**Fig. 4:**
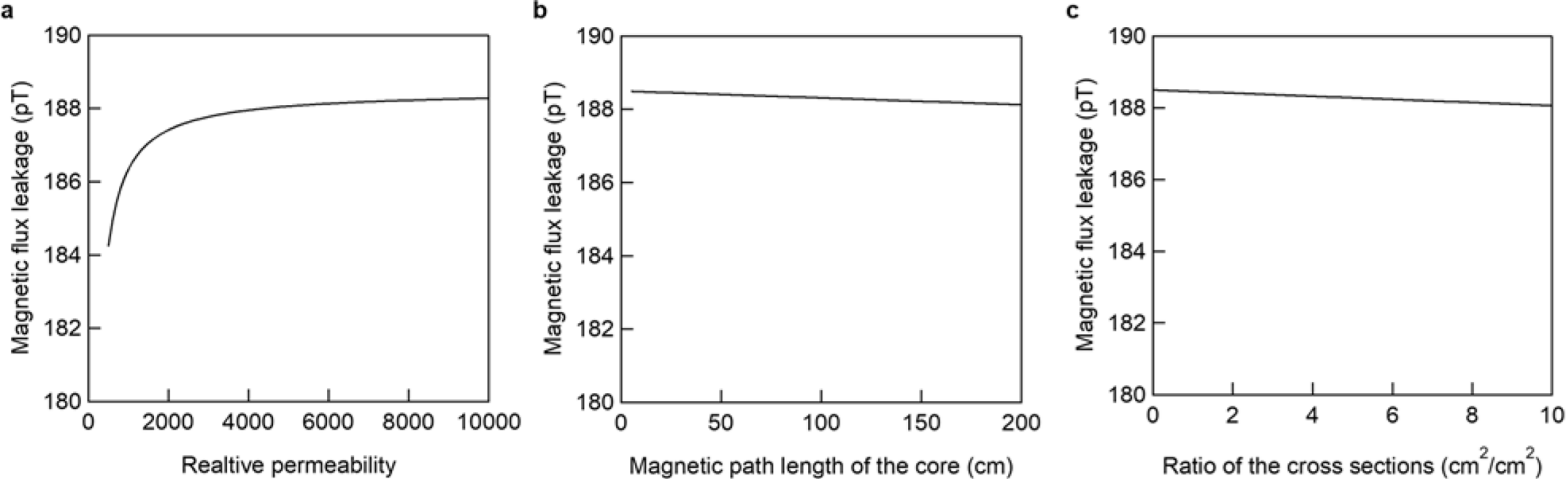
Simulated influence of key design parameters on the magnetic flux in the air gap. **a**, Magnetic flux as a function of the core relative permeability. The flux increases rapidly with permeability but saturates beyond $\:{\mu\:}_{\mathrm{r}}\approx\:3000$, including diminishing returns. **b**, Dependence of magnetic flux on the magnetic path length of the core. A shorter path length slightly increases the flux, suggesting a potential benefit for miniaturized designs. **c**, Effect of the ratio between the air gap and core cross-sectional areas, corresponding to tapering of the core edges. While the flux increase remains well below 1%, this parameter may still offer marginal performance optimization.

In summary, the DC ratio measurements yielded a ratio error of $\:150\:\text{ }\mathrm{n}\mathrm{A}/\mathrm{A}$ and an Allan deviation of $\:50\:\text{ }\mathrm{n}\mathrm{A}$, exceeding the performance of previously reported NV-based sensors under ambient conditions. These results confirm the suitability of the developed current comparator for precision current ratio metrology in both AC and DC regimes. Future work will focus on introducing a zero-flux compensation scheme and evaluating the long-term stability of the ratio error under varying environmental conditions. Improving magnetic and electrostatic shielding, optimizing the core structure—including the mitigation of material degradation caused by core cutting—and unifying the characterization in terms of ampere-turns will further consolidate the metrological foundation of this approach. In addition, direct comparisons with existing ACC and CCC will be essential for assessing the practical advantages and limitations of the proposed system.

**Table 2:** Uncertainty budget for the DC ratio measurement.

Source of uncertainty	Relative standard uncertainty
Type-A uncertainty (Allan deviation)	$\:5.0\times\:{10}^{-8}$
Magnetic flux measurement	$\:4.1\times\:{10}^{-8}$
Current source	$\:1.6\times\:{10}^{-10}$
Flux-current conversion coefficient	$\:1.2\times\:{10}^{-7}$
Combined standard uncertainty	$\:1.4\times\:{10}^{-7}$
Expanded uncertainty (* k=2*)	$\:2.7\times\:{10}^{-7}$

## Conclusion

We have demonstrated a compact, integrated current comparator capable of performing both AC and DC current ratio measurements with a type-A uncertainty on the order of $\:{10}^{-8}$, while the expanded uncertainty is on the order of $\:{10}^{-6}$ for AC and $\:{10}^{-7}$ for DC under ambient conditions by employing a NV diamond-based magnetometer. The developed system achieves a minimum detectable current of $\:5\times\:{10}^{-7}$ ampere turns, as verified through Allan deviation analysis in both AC and DC regimes. Unlike conventional current comparators—which typically rely on distinct architectures for AC and DC measurements, often requiring bulky windings or cryogenic environments—our approach offers a unified, cryogenics-free solution for high-sensitivity current ratio metrology.

Although tunnel-magnetoresistance (TMR) sensors are strong candidates for compact magnetic detection, the conversion process—magnetic flux to resistance and resistance to electrical current signal, requiring a calibration process. Together with this, their temperature dependence will make accurate zero-field and DC measurements challenging. In contrast, NV-diamond magnetometry provides a direct magnetic measurement, converting magnetic flux into a frequency shift through the gyromagnetic ratio, a fundamental physical constant. Moreover, by simultaneously tracking the two Zeeman-split ODMR resonances, the method inherently suppresses temperature-induced shifts in the zero-field splitting²⁴, resulting in strong resilience against temperature variations around the CC.

The present current comparator therefore represents a promising route toward a simplified and unified current-ratio calibration platform. While the proposed current comparator demonstrates high precision, further work is needed to evaluate its long-term stability, ratio-error repeatability, and robustness across different operating conditions. Future efforts will focus on implementing a zero-flux compensation scheme, improving magnetic and electrostatic shielding, and optimizing the core structure—particularly addressing the material issues associated with core cutting. In addition, expressing the system performance in terms of ampere-turns and comparing it directly with existing ACC, CCC, and TMR-based technologies will be essential for establishing its metrological advantages.

These advancements will not only enhance the performance of current ratio measurements in electric power standards but will also expand the applicability of the system to precision DC resistance bridges and fundamental quantum electrical standards. Ultimately, this work would open up the way for compact, cryogenics-free instrumentation capable of supporting a broad range of metrological and industrial applications.

## Methods

### Equivalent magnetic circuit model of current comparator

This section presents the theoretical framework for estimating the required sensitivity of the diamond magnetometer, supporting the design considerations discussed in the main text. To assess whether the diamond magnetometer satisfies the requirements of the current comparator, the residual magnetic flux in the air gap—measured by the magnetometer is estimated using a magnetic equivalent circuit model of the current comparator.

The current comparator with an air gap can be approximated as a series circuit consisting of magnetomotive forces (MMFs) generated by currents in the primary and secondary ratio windings (in opposite directions), along with the reluctances of the magnetic core and the air gap. The magnetomotive forces can be represented as $\:{N}_{\mathrm{P}}{I}_{\mathrm{P}}$, $\:{N}_{\mathrm{S}}{I}_{\mathrm{S}}$, where $\:{N}_{\mathrm{P}}$ and $\:{N}_{\mathrm{S}}$ are the number of turns of ratio windings, and $\:{I}_{\mathrm{P}}$ and $\:{I}_{\mathrm{S}}$ are the currents in the windings, respectively. The reluctances of the core and the air gap $\:{R}_{\mathrm{c}}$, $\:{R}_{\mathrm{g}}$ can be expressed as follows:

1 $$R_{{\mathrm{c}}} = \frac{{L_{{\mathrm{c}}} }}{{\mu _{0} \mu _{{\mathrm{r}}} S_{{\mathrm{c}}} }},R_{{\mathrm{g}}} = \frac{{L_{{\mathrm{g}}} }}{{\mu _{0} S_{{\mathrm{g}}} }}$$

where $\:{\mu\:}_{0}$ is the permeability in vacuum, $\:{\mu\:}_{\mathrm{r}}$ is the relative permeability of the magnetic core, $\:{S}_{\mathrm{c}}$, $\:{S}_{\mathrm{g}}$ are the cross sections of the core and the air gap, respectively. $\:{L}_{\mathrm{c}}$, $\:{L}_{\mathrm{g}}$ are the length of the magnetic paths of the core and the air gap, respectively. Since a one-to-one current comparator was developed in the present study, the number of turns of the ratio windings are equivalent: $\:N={N}_{\mathrm{P}}={N}_{\mathrm{S}}$, and the magnetomotive forces can be replaced with *NΔ I*, where $\Delta I = I_{{\mathrm{P}}} - I_{{\mathrm{S}}}$. Therefore, the magnetic flux in the air gap $\:\phi\:$ can be derived as:

2 $$\:\:\phi = \frac{{N_{{\mathrm{P}}} I_{{\mathrm{P}}} - N_{{\mathrm{S}}} I_{{\mathrm{S}}} }}{{R_{{\mathrm{c}}} + R_{{\mathrm{g}}} }} = \frac{{\mu _{0} \mu _{{\mathrm{r}}} S_{{\mathrm{c}}} S_{{\mathrm{g}}} N\Delta I}}{{S_{{\mathrm{g}}} L_{{\mathrm{c}}} + \mu _{{\mathrm{r}}} S_{{\mathrm{c}}} L_{{\mathrm{g}}} }}$$

**Fig. 5:**
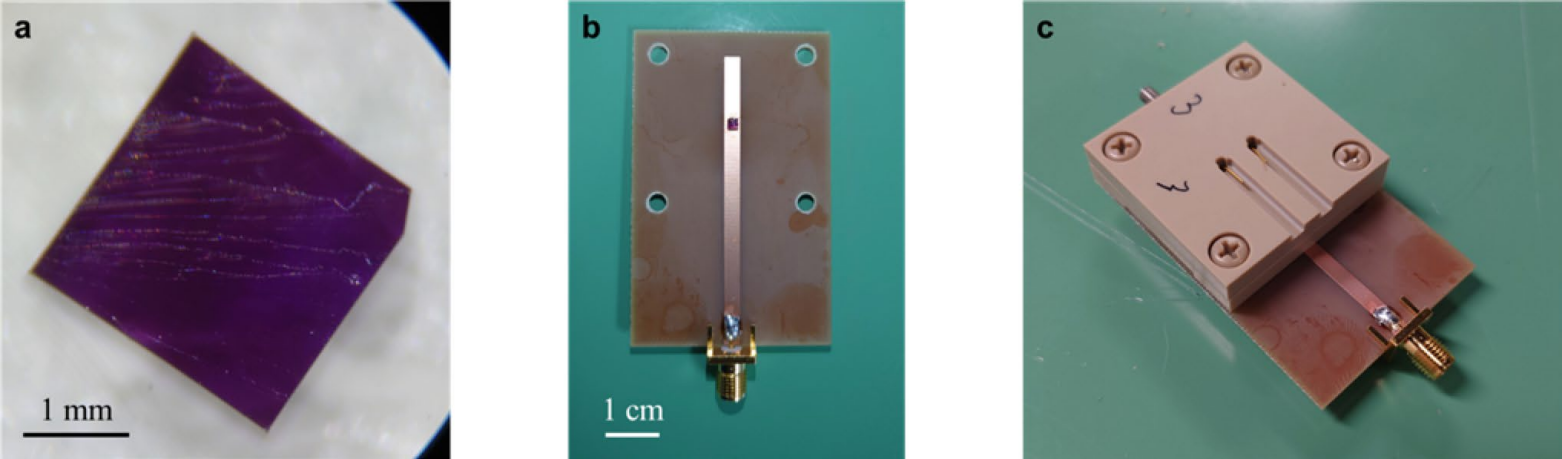
Photographs of the components used in the NV-based diamond magnetometer. **a**, A (111)-oriented type-Ib diamond chip synthesized by the high-pressure, high-temperature (HPHT) method and irradiated with electron beam to increase the NV center density. **b**, A microstrip waveguide resonant at $\:2.87\:\text{ }\mathrm{G}\mathrm{H}\mathrm{z}$, used for microwave excitation of the NV spin states. The diamond chip is mounted near the center of the waveguide line. **c**, Assembled sensor head, where the diamond chip is sandwiched between the microwave waveguide and the photodiode, which is covered with an optical long-pass filter. The total width of the sensor head is approximately $\:1\:\text{ }\mathrm{c}\mathrm{m}$, allowing its placement in the $\:2\:\text{ }\mathrm{c}\mathrm{m}$ air gap of the magnetic core.

Consequently, assuming that the magnetic flux was uniformly distributed in the air gap, the magnetic flux density * B* can be expressed as follows:

3 $$B\: = \frac{\phi }{{S_{{\mathrm{g}}} }} = \frac{{\mu _{0} \mu _{{\mathrm{r}}} N\Delta I}}{{\frac{{S_{{\mathrm{g}}} }}{{S_{{\mathrm{c}}} }}L_{{\mathrm{c}}} + \mu _{{\mathrm{r}}} L_{{\mathrm{g}}} }}$$

Replacing $\:{L}_{\mathrm{c}}$ with $\:{2\pi\:R-L}_{\mathrm{g}}$, where * R* is the radius of the magnetic path of the core finally yields the air-gap flux density * B* corresponding to a required sensitivity for the NV-diamond magnetometer:

4 $$B = \frac{{\mu _{0} \mu _{{\mathrm{r}}} N\Delta I}}{{2\pi R\frac{{S_{{\mathrm{g}}} }}{{S_{{\mathrm{c}}} }} + \left( {\mu _{{\mathrm{r}}} - \frac{{S_{{\mathrm{g}}} }}{{S_{{\mathrm{c}}} }}} \right)L_{{\mathrm{g}}} }}$$.

Among the parameters in the derived formula, the length of the air gap plays a critical role in the design of the current comparator, as highlighted in the main text. In contrast, the other parameters have only a minor influence on the magnetic flux within the air gap.

Magneti flux leakage increases with the relative permeability of the core material; however, this increase quickly saturates, as shown in Fig. 4a. Therefore, the magnetic core should possess moderately high permeability, while also being chosen based on other practical considerations such as manufacturability.

Similarly, reducing the length of the magnetic path of the core leads to a slight increase in the magnetic flux through the air gap, as illustrated in Fig. 4b. This suggests that minimizing the magnetic core is feasible without significantly compromising sensitivity.

Additionally, tapering the core to increase the air-gap-to-core area ratio results in only a sub-percent increase in magnetic flux (Fig. 4c).

### NV-diamond magnetometer design

This section describes the design of the sensor head for the diamond magnetometer, which is intended to be placed in the air gap of the current comparator. An initial prototype of the diamond sensor head was previously demonstrated at the CPEM2024 conference, confirming the feasibility of integrating an NV-diamond magnetometer into a magnetic core for current comparison.

A commercially available (111)-oriented type-Ib diamond, synthesized using the high-pressure, high-temperature (HPHT) method, was used (Fig. 5a). To increase the density of NV centers, the diamond chip was irradiated with an electron beam at a fluence of $\:3\times\:{10}^{18}\:\left(\mathrm{e}\mathrm{l}\mathrm{e}\mathrm{c}\mathrm{t}\mathrm{r}\mathrm{o}\mathrm{n}\left(\mathrm{e}\right)/{\mathrm{c}\mathrm{m}}^{2}\right)$ at an acceleration energy of $\:2\:\text{ }\mathrm{M}\mathrm{e}\mathrm{V}$, followed by annealing for 2 h at $\:1000\:\text{ }℃$. A microstrip line, resonant at approximately $\:2.87\:\text{ }\mathrm{G}\mathrm{H}\mathrm{z}$—the frequency corresponding to the zero-field splitting—served as a microwave antenna for spin-state manipulation (Fig. 5b). An optical long-pass filter with a cut-off of $\:610\:\text{ }\mathrm{n}\mathrm{m}$ was used to block background light including an excitation green laser while transmitting red fluorescence to the photodiode. The excitation laser is introduced from one of the side faces of the diamond chip and configured to excite a visually pale region where the sensor exhibited higher magnetic sensitivity. The sensor head was constructed by sandwiching the (111)-oriented diamond chip between the microwave waveguide and the photodiode covered by the optical filter (Fig. 5c). This compact structure, with a width of $\:1\:\text{ }\mathrm{c}\mathrm{m}$, allowed the sensor head to be placed in the $\:2\:\text{ }\mathrm{c}\mathrm{m}$ air gap of the magnetic core.

### Conversion coefficient for current and magnetic flux

**Fig. 6:**
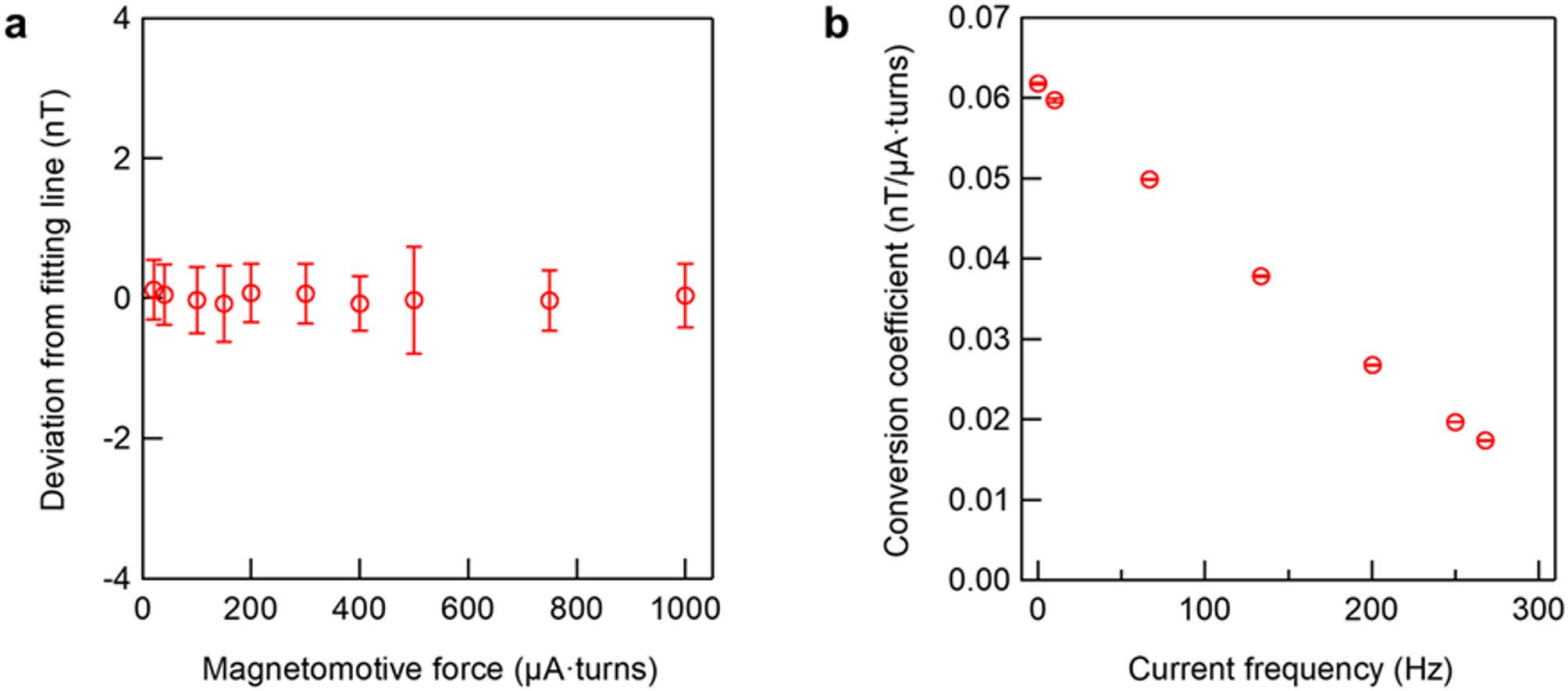
The calibration of conversion coefficients between input current and magnetic flux. **a**, Deviation between the fitted line and measured magnetic flux as a function of input current at $\:67\:\text{ }\mathrm{H}\mathrm{z}$. The error bars represent the standard deviation of the mean obtained from 100 times measurements. **b**, Conversion coefficient as a function of frequency from DC to $\:268\:\text{ }\mathrm{H}\mathrm{z}$. Each coefficient was derived from a linear fit at each frequency, and the error bars represent the error of the fit.

This section explains the analysis in Fig. 2a of the main text, detailing how the conversion coefficient between input current and magnetic flux was determined. This coefficient is essential for translating the measured magnetic flux in the air gap into an equivalent current difference, which is then used to calculate the ratio error of the current comparator. A linear relationship was established between the magnetic flux measured in the air gap and the input current applied to the primary winding at 67 Hz (Fig. 6). The slope of the linear regression yields a conversion coefficient of $\:0.498\:\text{ }\mathrm{n}\mathrm{T}/{\upmu\:}\mathrm{A}$, representing the proportionality between the measured magnetic flux and the amplitude of the input current at 67 Hz. Each point represents the deviation between the fitted line and measured magnetic flux obtained by 100 times measurements, and the error bars represent the standard deviation of the mean (Fig. 6a).

Since frequency dependence was observed during the experiments, conversion coefficients were determined at each frequency point—including DC—as shown in Fig. 6b. In Fig. 6b, the error bars, which represent the fitting error, were smaller than the markers.

## Supplementary Information

Below is the link to the electronic supplementary material.

Supplementary Material 1

## Data Availability

The data that supports the findings of this study are available from the corresponding authors upon reasonable requests.

## References

[1] Moore, W. J. M. & Miljanic, P. N. *The Current Comparator* (Peter Peregrinus Ltd., 1988).

[2] Harvey, I. K. A precise low temperature DC ratio transformer. *Rev. Sci. Instrum.* **43**, 1623–1629. 10.1063/1.1685508 (1972).

[3] Miljanic, P. N. et al. The development of the current comparator, a high-accuracy A-C ratio measuring device. *Trans. AIEE Part. I: Commun. Electron.* **81**, 359–368. 10.1109/TCE.1962.6373151 (1962).

[4] Kusters, N. L. & Moore, W. J. M. The compensated current comparator; A new reference standard for current-transformer calibrations in industry. *IEEE Trans. Instrum. Meas.* **IM-13**, 107–114. 10.1109/TIM.1964.4313383 (1964).

[5] Drung, D. et al. Improving the stability of cryogenic current comparator setups. *Supercond Sci. Technol.* **22**, 114004. 10.1088/0953-2048/22/11/114004 (2009).

[6] Seppä, H. et al. Thin-film cryogenic current comparator. *IEEE Trans. Instrum. Meas.* **48**, 365–369. 10.1109/19.769602 (1999).

[7] Kusters, N. L. et al. A current comparator for the precision measurement of D-C ratios. *IEEE Trans. Commun. Electron.* **83**, 22–27. 10.1109/TCOME.1964.6539564 (1964).

[8] MacMartin, M. & Kusters, N. A self-balancing direct current comparator for 20 000 amperes. *IEEE Trans. Magn.* **1**, 396–402. 10.1109/TMAG.1965.1062960 (1965).

[9] Rietveld, G. et al. Accurate DC current ratio measurements for primary currents up to 600 A. *IEEE Trans. Instrum. Meas.* **64**, 3055–3061. 10.1109/TIM.2015.2434096 (2015).

[10] Marzano, M. et al. Metrological assessment of DC current comparator resistance bridges. *Measurement* **215**, 112858. 10.1016/j.measurement.2023.112858 (2023).

[11] Marzano, M. et al. On the calibration of DC resistance ratio bridges. *Measurement* **223**, 113664. 10.1016/j.measurement.2023.113664 (2023).

[12] Greenberg, Y. S. Application of superconducting quantum interference devices to nuclear magnetic resonance. *Rev. Mod. Phys.* **70**, 175–222. 10.1103/RevModPhys.70.175 (1998).

[13] Storm, J. H. et al. An ultra-sensitive and wideband magnetometer based on a superconducting quantum interference device. *Appl. Phys. Lett.* **110**, 072603. 10.1063/1.4976823 (2017).

[14] Xu, D. et al. Low-noise second-order gradient SQUID current sensors overlap-coupled with input coils of different inductances. *Supercond Sci. Technol.* **35**, 085004. 10.1088/1361-6668/ac7ae5 (2022).

[15] Grohmann, K. & Hechtfischer, D. Self-Calibration Cryo Current Comparators for AC applications. *IEEE Trans. Instrum. Meas.* **IM-33**, 91–96 (1984).

[16] Loubser, J. H. N. & van Wyk, J. A. Electron spin resonance in the study of diamond. *Rep. Prog Phys.* **41**, 1201–1248. 10.1088/0034-4885/41/8/002 (1978).

[17] Jelezko, F. & Wrachtrup, J. Single defect centres in diamond: A review. *Phys. Status Solidi A*. **203**, 3207–3225. 10.1002/pssa.200671403 (2006).

[18] Doherty, M. W. et al. The nitrogen-vacancy colour centre in diamond. *Phys. Rep.* **528**, 1–45. 10.1016/j.physrep.2013.02.001 (2013).

[19] Taylor, J. M. et al. High-sensitivity diamond magnetometer with nanoscale resolution. *Nat. Phys.* **4**, 810–816. 10.1038/nphys1075 (2008).

[20] Sekiguchi, N. et al. Diamond quantum magnetometer with DC sensitivity of sub-10 pT Hz < sup > – 1/2 toward measurement of biomagnetic field. *Phys. Rev. Appl.* **21**, 064010. 10.1103/PhysRevApplied.21.064010 (2024).

[21] Barry, J. F. et al. Sensitive AC and DC magnetometry with nitrogen-vacancy-center ensembles in diamond. *Phys. Rev. Appl.* **22**, 044069. 10.1103/PhysRevApplied.22.044069 (2024).

[22] Schmitt, S. et al. Submillihertz magnetic spectroscopy performed with a nanoscale quantum sensor. *Science* **356**, 832–837. 10.1126/science.aam5532 (2017). 28546208 10.1126/science.aam5532

[23] Hatano, Y. et al. High-precision robust monitoring of charge/discharge current over a wide dynamic range for electric vehicle batteries using diamond quantum sensors. *Sci. Rep.* **12**, 13991. 10.1038/s41598-022-18106-x (2022). 36068253 10.1038/s41598-022-18106-x PMC9448744

[24] Hatano, Y. et al. Simultaneous thermometry and magnetometry using a fiber-coupled quantum diamond sensor. *Appl. Phys. Lett.* **118**, 034001. 10.1063/5.0031502 (2021).

[25] Muramatsu, H. et al. Diamond quantum magnetic sensor toward a precision comparison of currents with a current comparator. *Proc. IEEE*. **1-2**10.1109/CPEM61406.2024.10646090 (2024).

[26] Ma, D. et al. Magnetic noise calculation of mu-metal shields at extremely low frequencies for atomic devices. *J. Phys. D: Appl. Phys.* **54**, 025004. 10.1088/1361-6463/abbe4b (2021).

[27] Joint Committee for Guides in Metrology. Evaluation of measurement data—Guide to the expression of uncertainty in measurement (GUM 1995 with minor corrections). (2008).

[28] Lönard, D. et al. Limits of absolute vector magnetometry with NV centers in diamond. arXiv:2504.20750v2 (2025).

[29] Shi, Z. Y. et al. Current sensor based on diamond nitrogen-vacancy color center. *Chin. Phys. B*. **32**, 070704. 10.1088/1674-1056/acc3fe (2023).

[30] Liu, Q. et al. Millimeter-scale temperature self-calibrated diamond-based quantum sensor for high-precision current sensing. *Adv. Quantum Technol.* **6**, 2300210. 10.1002/qute.202300210 (2023).

[31] Liu, Q. et al. Closed-loop diamond quantum sensor for large range and high precision current measurement. *IEEE Sens. J.* **24**, 4356–4364. 10.1109/JSEN.2023.3348161 (2024).

