# Supplementary material for: Current comparator for both AC and DC ratio measurements with 10− 8-level type-a uncertainty
Source: Sci Rep. 2026 Aug 11;16:24158. doi: 10.1038/s41598-026-58868-2 (PMC13462737; doi:10.1038/s41598-026-58868-2)
Supplement: Supplementary file 1 [file 41598_2026_58868_MOESM1_ESM.docx]

**Supplementary Information**

This Supplementary Information provides additional experimental and theoretical details supporting the main text, including:

(1) a supplementary table, and

(2) Supplementary Note: comparisons with conventional technologies.

**Table A1** Comparison of current comparator technologies: Reported accuracy and operational regimes of various current comparator technologies. The present work bridges AC and DC regimes at high precision without cryogenics.

**Supplementary Note: Comparison with other existing current comparator technologies**

Table A1 presents a comparative summary of current comparator technologies, emphasizing the advantages of the proposed NV-diamond-based approach in terms of bandwidth, accuracy, and operating temperature. Unlike conventional current comparators that rely on detection windings or superconducting quantum interference devices (SQUIDs), the developed comparator using NV centers in diamond achieves a type-A uncertainty at the ${10}^{-8}$ level, while the expanded uncertainty is at ${10}^{-6}$ to ${10}^{-7}$ level, for both AC and DC measurements—all while operating at room temperature.

Compared to ACCs, which typically offer ${10}^{-6}$-level accuracy at $50 \mathrm{Hz}$ or $60 \mathrm{Hz}$, the NV-diamond-based comparator demonstrates comparable overall accuracy. Moreover, considering only the type-A uncertainty, it shows an improvement of more than an order of magnitude. For DC measurements, although the overall accuracy of the present comparator does not yet surpass that of cryogenic current comparators (CCCs) and direct current comparators (DCCs), the achieved type-A uncertainty is comparable to these established systems, without the need for superconducting cryogenic operations.

The integration of a compact diamond magnetometer as the null detector enables miniaturization and eliminates the requirement for cryogenic environments, paving the way for practical, high-precision, and scalable current comparator designs.
